# Electroconvulsive therapy for obsessive-compulsive disorder: A retrospective study

**DOI:** 10.3389/fpsyt.2022.1040443

**Published:** 2022-11-09

**Authors:** Kun Li, Jiang Long, Wei Deng, Bochao Cheng, Jiaojian Wang

**Affiliations:** ^1^Mental Health Center and Psychiatric Laboratory, The State Key Laboratory of Biotherapy, West China Hospital of Sichuan University, Chengdu, Sichuan, China; ^2^Affiliated Mental Health Center & Hangzhou Seventh People’s Hospital, Zhejiang University School of Medicine, Hangzhou, Zhejiang, China; ^3^Department of Radiology, West China Second University Hospital of Sichuan University, Chengdu, China; ^4^State Key Laboratory of Primate Biomedical Research, Institute of Primate Translational Medicine, Kunming University of Science and Technology, Kunming, China

**Keywords:** obsessive-compulsive disorder, electroconvulsive therapy, efficacy, comorbidity, refractory

## Abstract

**Background:**

Chronic mental diseases such as obsessive-compulsive disorder (OCD) are associated with a high disability rate. Some patients still do not improve their symptoms even with adequate cognitive-behavioral therapy and drug treatment. In the treatment of OCD, electroconvulsive therapy (ECT) is not considered a neuromodulation modality with sufficient evidence.

**Objective:**

This retrospective study aimed to determine the efficacy and associated risk factors of ECT in OCD patients.

**Materials and methods:**

The study included 21 OCD patients who underwent ECT at a high-volume center in China between January 2009 and December 2020. The demographics and clinical characteristics of the patients were assessed using descriptive statistics. Based on Clinical Global Impressions–Improvement scale, patients were categorized into response and non-response groups. Clinical and demographic characteristics of two groups of patients were compared.

**Results:**

An analysis of 21 patients was conducted. In total, 12 patients (57.1%) responded to ECT, 11 patients (52.4%) reported side effects, and an average of 7 ECT sessions were administered. In terms of demographic, there was no statistically significant difference between the two groups. It is noteworthy that the non-response group reported more depression and schizophrenia related disorders comorbidities than the response group (χ^2^ = 6.252, *P* = 0.041).

**Conclusion:**

The effectiveness of ECT in treating OCD is limited, especially in patients with refractory symptoms. Comorbidity with other mental disorders may affect the efficacy of ECT.

## Introduction

It is a chronic and disabling mental illness called obsessive-compulsive disorder (OCD). Obsessions and compulsions are the most prominent symptoms. In the general population, lifetime prevalence is 2.3%, and 12-month prevalence is 1.2% (1). Further, OCD prevalence in China is 1.63% over a 12-month period, with significant economic and social burden (2). Compared to the general population, all-cause mortality is twice as high, and comorbid other psychiatric disorders can further increase the risk (3). Estimates suggest that up to 90% of individuals with OCD have a comorbid psychiatric disorder (4, 5).

Cognitive-behavioral therapy and serotonin reuptake inhibitors (SRIs) are recommended as first-line treatments for OCD by various treatment guidelines and expert consensus (6–8). It is estimated that about 40–60% of people with OCD do not achieve satisfactory results in spite of the availability of a variety of treatment options (9). It may be due to intolerable side effects of the drug, unsatisfactory efficacy, poor compliance, treatment resistance, and comorbidity (10–13). A neurosurgical technique known as deep brain stimulation by FDA approved can be used to treat refractory OCD (14). However, its cost, availability, and intrusiveness have severely limited its use. It is therefore urgent to find alternative treatment strategies.

Electroconvulsive therapy (ECT) is a technique used to treat mental illnesses by applying electrical current through the brain. There is ample clinical evidence that ECT is mainly used in major depressive disorder (MDD), bipolar disorder, schizophrenia (15–18). However, Current guidelines do not include ECT as an alternative treatment for refractory OCD (6). The primary cause is an absence of sufficient evidence-based data (11, 19). Recent years have seen an increase in interest in ECT as a treatment for OCD. A systematic review included 265 patients and found reported a response rate of 60.4% (20). However, there were mainly case reports, no RCT test, and a small number of observational studies. As a result, clinical evidence regarding the use of ECT in OCD needs to be provided.

As a result, this retrospective study aimed to investigate ECT’s response rate among OCD patients and examine features associated with the response of OCD to ECT.

## Materials and methods

### Design and population

This study included patients who received ECT therapy at West China Hospital of Sichuan University between January 2009 and December 2020. The OCD patients were included if they met the criteria outlined in the International Classification of Diseases, Tenth Revision (ICD-10) for the diagnosis. We analyzed only the first ECT series of patients who received more than one treatment series. Consequently, 21 OCD patients were included in the study.

In accordance with the Declaration of Helsinki, it was approved by the Ethics Committee on Biomedical Research of West China Hospital of Sichuan University. Informed consent was not required for this study since it was exempt from institutional review.

### Electroconvulsive therapy treatment protocol

The ECT was performed with Thymatron System IV (SOMATICS, LLC). As part of our study, we collected data on ECT treatments, including the number of treatments and electrode placement. Whether or not ECT was required and the ECT protocol was determined by the clinical psychiatrist according to the patient’s condition. The diagnosis, treatment options, and risks and benefits of ECT were explained to patients and their legal guardians and informed consent was obtained prior to the start of ECT.

Typically, ECT administration was performed 3 times per week (on alternate days). The electrodes were positioned bilaterally in the temporal region. Under the supervision of an anesthesiologist and psychiatrist, propofol and succinylcholine were given separately for anesthetic and muscle relaxant.

### Clinical assessment

Demographic information was collected, including age, gender, and education level. In this study, we collected clinical factors, such as the age at which OCD onset occurred, the course of illness, comorbidities, and treatment histories. Furthermore, we examined ECT-related characteristics, such as the number of ECT sessions and the reasons for administering ECT.

We evaluated the charts independently by two authors (KL and JL) to determine their response to ECT. There was a consensus reached by reviewing disagreements with the experts (WD). Improvement of OCD were assessed using the Clinical Global Impressions-Improvement scale (CGI-I). On the CGI-I, a response was defined as a score of 1 (very much improved) or 2 (much improved). Patients or psychiatrists will record all side effects reported spontaneously or observed. Records and CGI-side effects scales were used to assess side effects.

### Statistics

All statistical analyses were conducted using SPSS (IBM SPSS, version 25, Chicago, IL, USA). We performed descriptive statistical analyses on all variables, including demographic characteristics, clinical factors, and ECT-related characteristics. Descriptive statistics involves computing means, standard deviations, and frequencies for continuous and categorical variables. Comparisons between groups were made with independent samples *t*-tests or Mann–Whitney *U* test for continuous variables. For categorical variables, we used the Chi-square test or Fisher’s exact test. The significance of the results was defined as *P* values < 0.05.

## Results

Twenty-one OCD patients receiving ECT were included in the study. Table 1 provides information about the demographics and clinical characteristics of the 21 participants. The average age was 27.14, the standard deviation was 10.41, the median age was 23, the minimum age was 16, the maximum age was 52. As for education levels, 95.2% of the respondents graduated from middle school or above. There are 11 (52.4%) males and 10 (47.6%) females. Concerning the clinical factors, the mean Course of illness was 82.00 ± 73.10 years, and the age of OCD onset was 20.82 ± 8.07 years. As far as comorbid conditions are concerned, 7 patients (33.3%) had comorbid MDD, 2 patients (9.5%) comorbid schizophrenia related disorders, and 12 (57.1%) had no comorbid condition at the time of admission. Of 21 patients, ECT was prescribed for severe OCD symptoms or major depression with suicidality in 4 (19.0%); treatment resistance in 9 (42.9%); catatonia symptoms in 1 (4.8%); psychosis, or agitation in 3 (14.3%). Seventeen (71.4%) patients were treated with adequate dose and time of SRIs. The OCD patients in this category are also called refractory OCD patients. There was a total of 21 ECT sessions, with an average (SD) of 7.00 (2.15) sessions per patient. After the ECT treatment course, the overall response rate was 57.1%. Side effects included headaches and amnesia in 5 patients (23.8%) respectively, while 11 patients (52.4%) did not report any side effects (see Table 1).

**TABLE 1:** Clinical and demographic characteristics.

Characteristic	Value (*n* = 21)
Age (years)	27.14 ± 10.41
**Education level**	
Primary school	1 (4.8)
Junior high school	5 (23.8)
High school	8 (38.1)
College	7 (33.3)
**Gender**	
Male	11 (52.4)
Female	10 (47.6)
Age of OCD onset (years)	20.82 ± 8.07
Course of illness (months)	82.00 ± 73.10
**Comorbidity patterns**	
None	12 (57.1)
Major depressive disorder	7 (33.3)
Schizophrenia related disorders	2 (9.5)
**Reasons for ECT administration**	
Treatment resistance	9 (42.9)
Severe OCD symptoms	4 (19.0)
Major depression with suicidality	4 (19.0)
Catatonia symptoms	1 (4.8)
Psychosis, or agitation	3 (14.3)
**Treatment history**	
Without adequate dose and time	6 (28.6)
With adequate dose and time	15 (71.4)
Number of ECT sessions	7.00 ± 2.15
Response	12 (57.1)
**Side effects**	
None	11 (52.4)
Headache	5 (23.8)
Amnesia	5 (23.8)
Muscle soreness	1 (4.8)

All variables are presented as “mean ± standard deviation” or “number (percentage).” ECT, electroconvulsive therapy; OCD, obsessive-compulsive disorder.

As shown in Table 2, the demographics and clinical characteristics of the responders and non-responders are compared. There were no significant differences in the demographic and clinical characteristics of the two groups of patients enrolled. In terms of comorbidities, the *p* value for comparison between the two groups was 0.041 (χ^2^ = 6.252). In addition, we separately compared the effects of comorbid MDD group with comorbid schizophrenia related disorders group and found no statistically significant difference between the two groups (χ^2^ = 0.735, *P* = 1). The comorbidity rate was higher in the non-response group (77.8%) than in the response group (16.7%).

**TABLE 2:** Comparison of clinical characteristics between response and non-response groups.

Variable	Response group (*n* = 12)	Non-response group (*n* = 9)	*T*, *Z* or χ^2^	*P*-value
Age	28.75 ± 11.23	25.00 ± 9.41	–0.810	0.428
**Education level**			5.775	0.147
Primary school	1 (8.33)	0 (0)		
Junior high school	4 (33.3)	1 (11.1)		
High school	2 (16.7)	6 (66.7)		
College	5 (41.7)	2 (22.2)		
**Gender**			1.289	0.387
Male	5 (41.7)	6 (66.7)		
Female	7 (58.3)	3 (33.3)		
Age of OCD onset (years)	23.00 ± 10.67	20.11 ± 8.46	–0.668	0.512
Course of illness (months)	69.00 ± 57.24	80.78 ± 70.55	0.423	0.677
**Comorbidity patterns**			**6.252**	**0.041**
None	10 (83.3)	2 (22.2)		
Major depressive disorder	2 (16.7)	5 (55.6)		
Schizophrenia related disorders	0 (0)	2 (22.2)		
**Reasons for ECT administration**			6.696	0.151
Treatment resistance	4 (33.3)	5 (55.6)		
Severe OCD symptoms	4 (33.3)	0 (0)		
Major depression with suicidality	2 (16.7)	2 (22.2)		
Catatonia symptoms	0 (0)	1 (11.1)		
Psychosis, or agitation	2 (16.7)	1 (11.1)		
**Treatment history**			2.353	0.178
Without adequate dose and time	5 (41.7)	1 (11.1)		
With adequate dose and time	7 (58.3)	8 (88.9)		
**Electrical parameters**				
Number of ECT sessions	7.75 ± 1.48	6.89 ± 1.83	–1.190	0.249
Energy (J)	25.06 ± 10.42	22.89 ± 6.03	–0.293	0.770
Stimulus duration (s)	5.23 ± 1.88	5.37 ± 1.63	–0.077	0.939
Current (mA)	872.50 ± 81.25	887.78 ± 71.90	–0.723	0.470
**Antidepressants**			3.257	0.298
SSRIs	5 (41.7)	7 (77.8)		
SNRIs	5 (41.7)	2 (22.2)		
TcAs	2 (16.7)	0 (0)		
Valproate	1 (8.3)	0 (0)	0.788	1
Benzodiazepines	4 (33.3)	4 (44.1)	0.270	0.673
**Antipsychotics**				
Olanzapine	0 (0)	1 (11.1)	1.400	0.429
Clozapine	0 (0)	2 (22.2)	2.947	0.171
Risperidone	0 (0)	2 (22.2)	2.947	0.171
Quetiapine	7 (58.3)	2 (22.2)	2.738	0.184
Aripiprazole	2 (16.7)	0 (0)	1.658	0.486
Palmitate	1 (8.3)	0 (0)	0.788	1
Sulpiride	2 (16.7)	1 (11.1)	0.130	1

All variables are presented as “mean ± standard deviation” or “number (percentage).” Comparing categorical variables were conducted using the Fisher exact test, and continuous variables were compared using the independent samples T-test or Mann–Whitney U test. ECT, electroconvulsive therapy; OCD, obsessive-compulsive disorder; SSRIs, selective serotonin reuptake inhibitors; SNRIs, serotonin and norepinephrine reuptake inhibitors; TCAs, tricyclic antidepressants. The bold values are statistically different.

## Discussion

To our knowledge, this is the first cohort of Chinese OCD patients treated with ECT. In this study, the effectiveness of ECT treatment of OCD and the occurrence of side effects were examined and predictors of efficacy were identified. In the total sample, at least 57.1% of patients reported positive responses to ECT. The OCD is frequently comorbid with MDD or schizophrenia-related disorders, with a 33.3 and 9.5% comorbidity rate, respectively. The effectiveness of ECT may be reduced if it is comorbid with other mental disorders, which is notable.

While this study suggests ECT has 57.1% response rate for OCD, patients are highly heterogeneous. The results of this study were similar to previous observational studies in which ECT significantly improved symptoms of OCD in most patients (21). It might be associated with an increase in paroxetine binding sites in the frontal cortex caused by ECT (22). Furthermore, previous studies have demonstrated that the therapeutic effects of ECT may have been partly related to brain-derived neurotrophic factor (23) and neuro metabolites (13). There were 21 patients with OCD, and 15 (71.4%) had received adequate dose and time of SRIs. The remaining 6 patients (28%), however, did not receive adequate treatment before ECT and were not classified as OCD patients with refractory symptoms. A systematic review included only 265 patients, with a response rate of 60.4%, but only 52.7% of these patients had been treated with SRIs and 16.9% had received a full dose (20). In clinical practice, ECT may be prematurely administered to some patients. Furthermore, 8 of the non-responders (88.9%) and 7 of the responders (58.3%) had refractory OCD. Due to this, ECT has a certain effect on OCD patients, but it has a less significant effect on refractory patients. The use of ECT has also been associated with significant improvements in refractory OCD in many cases (24–31). A small prospective study also found that ECT had an anti-obsessional effect (32). So, in patients with refractory OCD, ECT remains recommended.

It is interesting to note that 13 (61.9%) patients who received ECT had refractory or severe symptoms, but other patients required ECT for comorbidities of other severe psychiatric symptoms that needed to be addressed. It is noteworthy that patients with OCD who have comorbid MDD or schizophrenia-related disorders were less effective than those without. The results of this study are consistent with previous studies (20). The obsessive-compulsive symptoms changed independently of depressive symptoms (21, 33). The neural circuits of OCD with depression comorbidity differ from those of other depressed patients, according to some studies (34). OCD is markedly different from depression in that it is insensitive to norepinephrine reuptake inhibitors (35). There may be no link between serotonergic function and ECT’s antidepressant properties (36), but it is important to ECT’s anti-obsessive-compulsive effects. The ECT, although effective in depression, seems to affect the hypothalamus, insula (37), and hippocampus (38, 39), but in OCD, it affects the cortico-striato-thalamo-cortical circuits (40–45). The mechanism of action of ECT in treating OCD may be related to the inhibition in the prefrontal cortex (46). Therefore, treating obsessive-compulsive symptoms may be affected by the complex neurologic mechanisms involved in OCD comorbidities. Another factor influencing the efficacy of ECT for OCD comorbid depression may be the therapeutic target. A single stimulation target does not provide an optimal plan for individualized treatment, especially for the complex brain network disorders of OCD (43, 47). Future research needs to combine multiple approaches to achieve precise stimulation, such as neuronavigation (48).

Moreover, ECT has been found to be effective in treating both obsessive-compulsive symptoms and depression symptoms (13, 21, 27, 49, 50). It is necessary to conduct studies with larger samples in order to confirm this conclusion. Even in the presence of comorbidities, ECT is still recommended for patients with refractory OCD because of the invasiveness and cost of neurosurgery.

It is also important to note that there are some limitations to this research. Due to the retrospective nature of this study, we were unable to control for various confounding variables. The future will demand large-scale, high-quality randomized controlled studies to fulfill clinical practice requirements. Secondly, statistical power was limited by the small sample, which may explain some of the non-significant findings. Moreover, we lack quantifiable metrics for comorbid symptoms, such as catatonic symptoms (51–53). As the study population was heterogeneous, future research will need to focus specifically on patients who are refractory to treatment for OCD.

## Conclusion

As a result of this study, we can conclude that ECT for OCD has limited efficacy, especially in refractory patients. There is a possibility that comorbid MDD or schizophrenia related disorders could adversely affect ECT. Therefore, this study needs to be further verified by a randomized controlled trial involving a large sample size.

## Data availability statement

The raw data supporting the conclusions of this article will be made available by the authors, without undue reservation.

## Ethics statement

The studies involving human participants were reviewed and approved by Ethics Committee on Biomedical Research of West China Hospital of Sichuan University. Written informed consent for participation was not required for this study in accordance with the national legislation and the institutional requirements.

## Author contributions

KL: conceptualization, formal analysis, data curation, and writing – original draft. JL: formal analysis and data curation. BC: data curation, investigation, and methodology. JW: data curation, project administration, supervision, and writing – review and editing. WD: conceptualization, data curation, project administration, supervision, and writing – review and editing. All authors contributed to the article and approved the submitted version.
